# A Novel C-Type Lectin Receptor-Targeted α-Synuclein-Based Parkinson Vaccine Induces Potent Immune Responses and Therapeutic Efficacy in Mice

**DOI:** 10.3390/vaccines10091432

**Published:** 2022-08-30

**Authors:** Sabine Schmidhuber, Sandra Scheiblhofer, Richard Weiss, Mihály Cserepes, József Tóvári, Gabriele Gadermaier, Erwan Bezard, Francesca De Giorgi, Francois Ichas, Dirk Strunk, Markus Mandler

**Affiliations:** 1 Tridem Bioscience GmbH & CoKG, Campus Vienna Biocenter, Dr.-Bohrgasse 7, 1030 Vienna, Austria; 2 Department of Biosciences and Medical Biology, Paris Lodron University Salzburg, Hellbrunner Straße 34, 5020 Salzburg, Austria; 3 KINETO Lab Ltd., Csillaghegyi út 19-21, H-1037 Budapest, Hungary; 4 Motac Neuroscience, Alderley Park, Macclesfield SK10 4TF, UK; 5 Institut des Maladies Neurodégénératives, UMR 5293, Université de Bordeaux, 33076 Bordeaux, France; 6 Cell Therapy Institute, Paracelsus Medical University Salzburg, 5020 Salzburg, Austria

**Keywords:** Parkinson’s disease, vaccine, vaccination strategy, α-synuclein, public health, cost effectiveness

## Abstract

The progressive accumulation of misfolded α-synuclein (α-syn) in the brain is widely considered to be causal for the debilitating clinical manifestations of synucleinopathies including, most notably, Parkinson’s disease (PD). Immunotherapies, both active and passive, against α-syn have been developed and are promising novel treatment strategies for such disorders. To increase the potency and specificity of PD vaccination, we created the ‘Win the Skin Immune System Trick’ (WISIT) vaccine platform designed to target skin-resident dendritic cells, inducing superior B and T cell responses. Of the six tested WISIT candidates, all elicited higher immune responses compared to conventional, aluminum adjuvanted peptide-carrier conjugate PD vaccines, in BALB/c mice. WISIT-induced antibodies displayed higher selectivity for α-syn aggregates than those induced by conventional vaccines. Additionally, antibodies induced by two selected candidates were shown to inhibit α-syn aggregation in a dose-dependent manner in vitro. To determine if α-syn fibril formation could also be inhibited in vivo, WISIT candidate type 1 (CW-type 1) was tested in an established synucleinopathy seeding model and demonstrated reduced propagation of synucleinopathy in vivo. Our studies provide proof-of-concept for the efficacy of the WISIT vaccine technology platform and support further preclinical and clinical development of this vaccine candidate.

## 1. Introduction

Synucleinopathies are neurodegenerative disorders characterized by a progressive accumulation of α-synuclein (α-syn) in the brain, including Parkinson’s disease (PD), Lewy body dementia (LBD), and multiple-system atrophy (MSA). The accumulation of α-syn is widely considered to be causal to the clinical manifestations of synucleinopathies by interfering with synaptic, mitochondrial, and lysosomal function in cortical and sub-cortical brain regions [1,2,3,4]. PD is the most common synucleinopathy, affecting an estimated 1 in 500 individuals [5]. Most people develop early symptoms of the disease in their fifties. Symptom severity generally increases with age. There are currently no disease-modifying therapies available, but both passive and active immunotherapeutic approaches have shown promise for potential disease-modifying treatment of synucleinopathies [6,7,8,9].

Passive immunization with monoclonal antibodies (Abs) has previously been shown to reduce neurodegeneration and α-syn accumulation [10,11,12], decrease cell-to-cell transmission of α-syn [13,14,15], and inhibit prion-like propagation of pathology [16,17] in numerous animal studies.

Several passive immunotherapies for PD are now in early clinical development [18,19]. However, similar to the development of amyloid beta (Aβ) immunotherapy for Alzheimer’s disease (AD), which resulted in a very low success rate in admitting such treatment modalities for clinical use [20,21,22], recent drawbacks to PD immunotherapy have also been reported. The anti-α-syn-directed monoclonal antibody Cinpanemab has shown highly promising preclinical efficacy and selectivity for potential pathologically relevant α-syn species [15]; however, development was halted due to lacking overall effects on clinical measures of disease progression and changes in DaT-SPECT imaging (Dopamine transporter single-photon emission computed tomography) [19]. Prasinezumab, a monoclonal antibody targeting aggregated α-syn, had no meaningful effect on global or imaging measures of Parkinson’s disease progression in a recent phase II study but generated positive signals on multiple secondary and exploratory end points [23]. Although disappointing, the negative data have not deterred Roche/Prothena from commencing a Phase 2b trial in early PD patients.

While these results may mark the end for passive immunotherapy in early-stage PD, success may yet be achieved with the same or similar agents in prodromal PD, or alternative mechanisms to affect aggregated α-syn may be beneficial. The latter may include vaccines targeting α-syn aggregates given the fact that the antibody response they induce is polyclonal. Furthermore, the epitope that is addressed within the α-syn molecule may be critical. Thus, it is highly important to further investigate and develop novel, adapted therapeutic or preventive immunotherapies to fight amyloidogenic diseases, especially α-syn. In addition, passive therapy approaches suffer from high production costs, side effects, and the need for frequent administration, limiting their scalability [24]. For these reasons, active immunization is considered to be the most viable therapy option at scale.

Active immunization based on α-syn has also been shown to improve α-syn clearance and prevent neurodegeneration in diverse animal studies [25,26,27,28,29]. Several pre-clinical programs are developing active immunotherapy for synucleinopathies, administered both intramuscularly (i.m.) and subcutaneously (s.c.). Qb-PD vaccine candidates use Qbeta virus-like particles conjugated to α-syn peptides and induced anti-α-syn Ab titers, but candidates have failed so far to significantly alter α-syn levels or PD symptoms in vivo [29]. Recently, Prothena Bioscience Inc. described conventional peptide protein conjugate vaccines consisting of *C*-terminal linear single and tandem α-syn peptides conjugated to the non-toxic mutant of diphtheria toxin CRM 197 and adjuvanted with the Quillaja Saponaria-derived saponin QS21 [30]. Antibodies induced by the tandem α-syn vaccines bound to pathogenic α-syn and showed inhibition of the uptake of soluble α-syn aggregates into cells.

Kim et al. [27] also described PD DNA-vaccines based on the MULTITEP platform. These candidate vaccines use promiscuous T helper cell epitopes attached to well-known α-syn derived B cell epitopes. In vivo results showed the induction of Abs specific to α-syn, which significantly reduced PD/DLB-like pathology, as well as neurodegeneration in transgenic mice overexpressing human α-syn [27]. In addition, two PD vaccines in early clinical development have been described: PD01A/ACI-7104 (AC Immune, Switzerland) and UB312 (Vaxxinity, USA). PD01A uses short peptides conjugated to keyhole limpet hemocyanin (KLH) and an aluminum oxyhydroxide adjuvant [25,26,28], and is administered subcutaneously. PD01A/ACI-7104 has completed phase I clinical trials with a promising safety profile and evidence of a humoral immune response lowering oligomeric α-syn levels in the cerebrospinal fluid (CSF) [31]. UB312 relies on the UBITh platform using helper T cell epitopes covalently linked to α-syn peptides to enhance immunogenicity. UB312 is adjuvanted with aluminum phosphate and a proprietary CpG oligodeoxynucleotide (AdjuPhos/CpG1) and is administered i.m. Initial results from a phase I trial in healthy volunteers indicated a dose-dependent induction of α-syn-specific Abs, which cross the blood–brain barrier [32]. Notably, dose titration in the phase IA trial had to be stopped prematurely for safety reasons. Currently, a phase IB trial in PD patients is ongoing.

One of the most prominent classes of polysaccharides used as alternative adjuvants is the class of C-type lectins (CLECs). Various CLECs including mannan [33] and *N*-acetylglucosamines [34,35], as well as the β-glucan family, have been proposed as alternative vaccine adjuvants [36,37,38]. Members of the β-glucan family such as β-(1,3)-D-glucans, Laminarin, and Curdlan, among others, constitute a class of carbohydrate pathogen-associated molecular patterns (PAMPs), with a strong adjuvant-like function. These PAMPs play a critical role in the initiation of innate and adaptive immune responses [39] and are recognized by the innate immune system, primarily by their cognate receptor dectin-1. The binding of β-glucans to dectin-1 activates phagocytosis, respiratory burst, and the secretion of cytokines, leading to the activation of antigen-presenting cells (APCs) and the migration of activated APCs to draining lymph nodes where a sustained immune response is initiated [36,37]. Glucan particles (GPs), highly purified 2–4 µm hollow porous cell wall microspheres composed of primarily β-(1,3)-D-glucans, have already been used as experimental encapsulation agents for different antigens and drugs. Recently, Rockenstein et al. showed that GPs loaded with recombinant human α-syn and rapamycin induced a robust neuroprotective humoral immune response and regulatory iTreg (CD25 and FOXP3+) cells leading to an alleviation of α-syn triggered pathologic alteration in a murine synucleinopathy model [40].

In contrast to large micro-particular encapsulation agents such as GPs, different β-glucans have also been used to generate nanoparticles consisting of protein-glucan neoglycoconjugates for dendritic cell (DC) targeting. Several studies have demonstrated that β-glucans can be used as potential adjuvants for vaccination using model antigens such as OVA [36,37], or fusion proteins based on MUC1 [41]. The immune response cascade that is triggered in response to the recognition of β-glucans, and their strong immunomodulatory characteristics, supports the potential use of β-glucans as DC-specific vaccine delivery agents and the generation of β-glucan-based neoglycoconjugate vaccines in other diseases, including PD.

Traditional vaccination approaches target antigen into the muscle or the subcutis, which are sparsely populated by immunocompetent cell types. Thus, such vaccines, including current PD vaccines, require the use of potent adjuvants. In contrast, novel approaches deliver vaccines directly into the skin, an organ rich in DC and other immune cell types. Intradermal (i.d.) vaccination has been previously shown to generate more efficient specific B- and T cell responses compared to conventional routes of application [42,43].

Alternative vaccines and/or adjuvants specifically designed for i.d. application therefore have the potential to generate a more effective immune response compared to existing vaccines. Here, we present the novel peptide-based vaccine platform WISIT (Win the Skin Immune System Trick) using different interchangeable linear α-syn peptides with a CLEC/β-glucan vaccine backbone for the development of an α-syn specific PD vaccine. WISIT constructs are unique as the backbone itself is the DC targeting/stimulating adjuvant (β-glucan), fused to variable T helper and B cell peptide epitopes. We provide proof-of-concept for the WISIT vaccine technology platform and demonstrate that candidates targeting human α-syn are superior to conventional-type conjugate vaccines in eliciting functional, protective immune responses in vitro and in vivo.

## 2. Materials and Methods

### 2.1. Generation of Conjugates

All peptides were synthesized by FMOC solid-phase peptide synthesis (EMC microcollections GmbH; Tübingen, Germany). Peptides used for the individual candidate vaccines are listed in Table 1. For WISIT vaccines, an activated CLEC solution was stirred with dissolved hydrazide-modified peptides in a coupling buffer (sodium acetate buffer at pH 5.4), and the carbohydrate concentration in conjugates was estimated using the anthrone method. Peptide concentration in WISIT vaccines was determined by amino acid analysis. All WISIT vaccines were applied to animals i.d., unless stated otherwise. For conventional vaccines, peptides were coupled to the carrier CRM-197 (EcoCRM™-Maleimide, Fina Biosolutions LCC, Rockville, MD, USA). Briefly, the excess peptide was added to EcoCRM™-Maleimide for coupling in a 200 mM Na-phosphate buffer (pH 6.8) and subsequently dialyzed 3× with PBS. Ellmann reagent 5,5′-dithio-bis-(2-nitrobenzoic acid; Thermo Fisher Scientific, Waltham, MA, USA) was used for quantifying free sulfhydryl groups in solution. The conventional CRM-197 conjugates were further formulated with aluminum (aluminum oxyhydroxide adjuvant 2%; InvivoGen, San Diego, CA, USA) and applied to animals s.c. Identical amounts of conjugated peptides were injected per mouse for any comparison of the CRM-197 vaccine to WISIT vaccine candidates.

### 2.2. Fc-Dectin-1 Binding

Fc-dectin-1 binding was analyzed by enzyme-linked immunosorbent assay (ELISA) using a soluble murine Fc-dectin-1a receptor (InvivoGen, San Diego, CA, USA) as described in Korotchenko et al. [37]. Briefly, ELISA plates were coated with a reference CLEC and conjugates were tested in a competitive ELISA to demonstrate dectin binding. Binding efficacy was calculated as IC_50_ (Half maximal inhibitory concentration) values with Prism^®^ 9.3 (GraphPad Inc, San Diego, CA, USA) by non-linear regression analysis (four-parameter logistic fit function).

### 2.3. Activation Analysis Using Bone Marrow-Derived Dendritic Cells

Immature mouse DC generated in vitro with GM-CSF were stimulated with either lipopolysaccharide (LPS) using the equivalent dose contained in WISIT-conjugate preparations, WISIT-conjugates, conventional conjugates (CRM197), or oxidized CLEC-only, for 24 h. WISIT and conventional conjugates and CLEC-only preparations were used in increasing doses starting from 62.5 µg/mL to 500 µg/mL of the respective sugar. For comparison, LPS has been used as a control starting at a concentration of 2 ng/mL. DCs were identified based on CD11b/CD11c expression, and the surface expression of CD80 and major histocompatibility complex (MHC) class II were analyzed. Preparations were measured by flow cytometry. The expression of activation markers was analyzed using FCS Express (version 7.14, De Novo Software), where the expression of WISIT- and conventional conjugate preparation-treated DCs was compared to the expression of LPS-treated DC.

### 2.4. Animal Experiments

Immunological assessment: Female BALB/c mice (*n* = 5–6 mice per group; age 8–10 weeks) were immunized three times once every two weeks and blood samples were taken one day before each vaccination and two weeks after the last application unless otherwise indicated. All animal procedures were conducted at Kineto Lab Ltd. (Budapest, Hungary) in compliance with national Hungarian legislation. Experiments were performed under approval number PE/EA/448-7/2021 and were performed according to HLASA recommendations for animal use.

### 2.5. Induction of Synucleinopathy in Mice

All experimental procedures were conducted at Motac neuroscience, Bordeaux, in accordance with the European Communities Council Directive (2010/63/EU) for the care of laboratory animals in an AAALAC-accredited facility. For the induction of synucleinopathy, nine-week-old male C57BL/6 mice were stereotactically injected at the level of the right substantia nigra, with preformed polymorph fibrils (PFFs; i.e., preformed sonicated τ-polymorph α-syn fibrils 1B). PFFs were prepared and validated as described previously [44]. Briefly, each animal received a unilateral injection of 2 µL PFFs 1B solution (concentration: 2.5 mg/mL) into the region immediately above the right substantia nigra (coordinates from bregma: −2.9 AP, ±1.3 L and −4.5 DV) at a flow rate of 0.4 µL/min [44], and the needle was left in place for 5 min before being slowly withdrawn from the brain.

Starting on the same day as the inoculation, animals received three i.d. immunizations, once every two weeks (i.e., weeks 0, 2, 4), with either CW-type 1 (*n* = 5) or non-conjugated CLEC (*n* = 10) as the control, followed by a boost immunization in week 10. Upon termination of the study (day 126), cerebrospinal fluid (CSF) was collected by cisterna magna puncture, and brains were carefully removed and fixed in paraformaldehyde (PFA; 4%). Coronal serial sections of the entire brain (from rostral cerebral cortex anterior to striatum to the medulla—i.e., bregma −6.72 mm) using a cryostat at 50 µm intervals were collected and processed for immunohistochemistry.

### 2.6. Immunohistochemistry (IHC)

IHC staining of phosphor-S129 α-synuclein (pS129 α-syn) on coronal serial sections was performed as previously described [44]. The monoclonal rabbit anti-pS129 α-syn antibody EP1536Y (ab51253, Abcam, Cambridge, UK) was used, followed by incubation with labelled polymer-HRP anti-rabbit (Dako EnVision+^TM^ Kit, K4011, Agilent, Santa Clara, CA, USA). Visualization of pS129 α-syn staining was performed with Dako DAB (K3468), and sections were counterstained with the Nissl stain. The actual number of pS129 α-syn aggregates per structure (cerebral cortex, striatum, thalamus, substantia nigra, and brainstem) and the total number of pS129 α-syn aggregates were assessed using whole-section acquisition by Panoramic Scan II (3DHISTECH, Hungary) and further processed with the ad-hoc developed QuPath algorithm.

### 2.7. Vaccine Antibody Titer Determination

Standard ELISA technology was used to measure levels of vaccine-induced antibodies in plasma. Briefly, ELISA plates (Nunc Maxisorb; Thermo Fisher Scientific, Waltham, MA, USA) were coated with recombinant human α-syn protein (1 µg/mL; Anaspec; Fremont, CA, USA) using 50 mM sodium carbonate buffer overnight at 4 °C. Plates were blocked with 1% bovine serum albumin, and plasma samples were serially diluted in the plates. The detection of target-specific antibodies was performed with biotinylated anti-mouse IgG (Southern Biotech, Birmingham, AL, USA) and a subsequent color reaction using Streptavidin-POD (Roche, Basel, Swiss) and TMB. Titers were calculated as EC_50_ values using Prism^®^ 9.3 following non-linear regression analysis (four-parameter logistic fit function).

### 2.8. Inhibition and Avidity ELISA

Inhibition ELISA was performed as previously described [45]. Briefly, titrated amounts of α-syn monomer and α-syn fibrils (both from Abcam, Cambridge, UK), pre-incubated with vaccine-candidates-induced Abs, were added to ELISA microtiter plates densely coated with α-syn monomers (Abcam, Cambridge, UK). The detection of Abs bound to the immobilized antigen was analyzed and IC_50_ values were calculated by Prism^®^ 9.3 following non-linear regression analysis (four-parameter logistic fit function).

Antibody avidity to α-syn filaments was determined in the presence of the chaotropic agent sodiumthiocyanate (NaSCN) by an adapted ELISA. Briefly, ELISA plates (Nunc Maxisorb; Thermo Fisher Scientific, Waltham, MA, USA) were coated with α-syn filaments (Abcam, Cambridge, UK) using 50 mM sodium carbonate buffer, overnight at room temperature (RT). Plates were blocked with 1% bovine serum albumin, and constant amounts of plasma samples were added and incubated for 1 h. Following plate washing, 100 μL of a chaotropic agent was added at increasing concentrations (from 0 to 3 M) for 20 min at RT. Plates were washed again, and residual antibody binding was determined. Absorbance values in the absence of NaSCN were taken as the total effective binding of the specific antibody (100% binding), and subsequent absorbance values in the presence of increasing concentrations of NaSCN were converted to the corresponding percentage of the total bound antibody. The data were fitted to a plot of (% binding) vs. (log) concentration of NaSCN, and linear regression analysis was used to estimate the avidity index. The avidity index was defined as the concentration necessary to decrease the initial absorbance by 50%.

### 2.9. Thioflavin T Assay for Aggregation

Monomeric α-syn samples (recombinant human α-syn protein monomer (active); Abcam, Cambridge, UK) were incubated with shaking at 1000 rpm at 37 °C in nonbinding polystyrene 96-well plates (Greiner Bio ONE, Frickenhausen, Germany). Next, sonicated preformed α-syn fibrils (sPFF; recombinant human α-syn protein aggregate (active); Abcam, Cambridge, UK) alone or together with vaccine-candidate-induced Abs, the control mouse IgG1 antibody, or untreated murine plasma were added. Titrating amounts ranging from 0 to 5 μg/mL of vaccine-candidate-induced Abs, the control mouse IgG1 antibody (Southern Biotech, Birmingham, AL, USA), or untreated murine plasma (in equivalent amount) were used. All results are mean values of at least triplicate samples.

### 2.10. Statistical Analysis

All statistical analyses were conducted using Prism^®^ 9.3. Data are presented as mean ± standard error of the mean (SEM) and were analyzed with one-way analysis of variance (ANOVA) with post-hoc Tukey’s multiple comparison test unless indicated otherwise. Linear regression analysis was applied to calculate different parameters. Pearson correlation analysis was used to assess the strength and direction of the linear relationships between pairs of variables. A value of *p* < 0.05 was considered statistically significant.

## 3. Results

### 3.1. WISIT Vaccine Design

**Figure 1:**
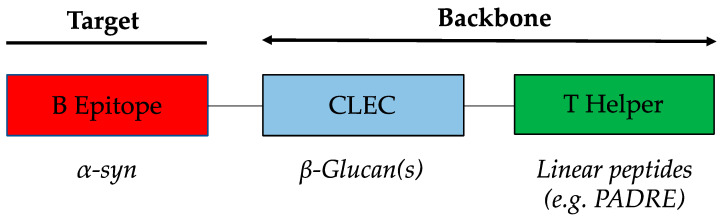
Functional building blocks of the α-syn WISIT vaccine. α-syn WISIT vaccines are neoglycoconjugates. The conjugate consists of three functional elements. Linear, pan-HLA T-cell epitopes (polypeptides), which have already been previously successfully used in humans (e.g., PADRE) provide T cell help. α-syn specific B-cell epitopes define the specificity of the antibody response induced. B-cell epitopes are 7–12-mer peptides (see Table 1). Both polypeptides are covalently coupled to the CLEC. Functionally, the unit of the CLEC and the T cell epitope is the vaccine’s backbone.

The WISIT platform technology is specifically designed to target skin-derived DCs. The skin plays a central role in triggering immune responses and defense against infections. The major advantage of targeting skin is that its reactory nature means that no additional adjuvants are required for vaccine application. Classical conjugate vaccines normally require a target-specific B cell epitope and a carrier protein providing foreign T cell epitopes in order to induce an efficient immune response. To ensure sufficient immunogenicity, adjuvants are added to classical conjugate vaccines, which are then administered i.m. or s.c. Although these tissues can mediate immune responses, they are not specialized in doing so. WISIT vaccines include a sugar residue (CLEC/β-glucan), which allows for the direct targeting of DC without the need for a standard adjuvant (Figure 1).

### 3.2. Production of WISIT Vaccine Candidates for α-Syn

To generate the reactive sugar backbone of the WISIT vaccines (Figure 1) for α-syn, CLECs/β-glucans were first activated by mild periodate oxidation. WISIT PD vaccine candidates CW-type 1–6 were produced by hydrazide-based coupling of six different B-cell epitope peptides (7 to 12-mers) derived from *N*- and *C*-terminal α-syn domains and the linear non-natural pan DR epitope (PADRE) as a T-helper epitope peptide, including a C-terminal hydrazide linker for coupling. The same B cell epitope peptides were coupled to CRM197 by maleimide–thiol coupling to provide conventional peptide-carrier conjugates used as benchmark controls (CC-type 1–6), respectively (Table 1).

### 3.3. WISIT Vaccine Candidates Targeting α-Syn Are Biologically Active in DCs

**Figure 2:**
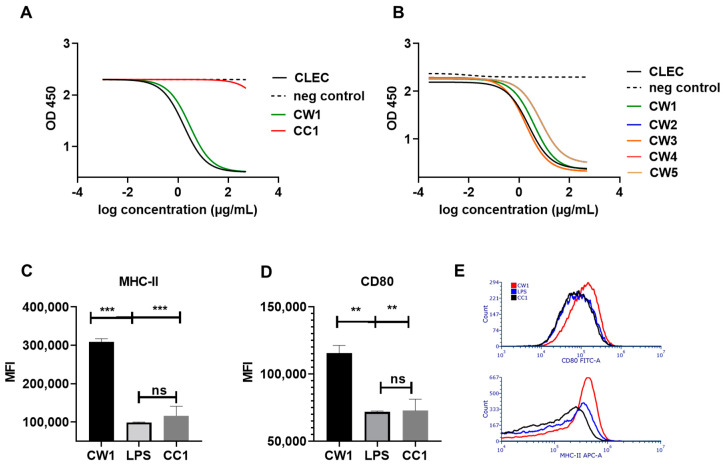
Dectin-1 binding and dendritic cell (DC)-activation by WISIT vaccines. (**A**) Comparative analysis of the dectin-1 binding ability determined by ELISA of WISIT-type (CW-type 1) and conventional, non-WISIT candidate 1 vaccine (CC-type 1) and (**B**) of different WISIT-type conjugates (CW-type 1–5), as well as CLEC alone, are shown. (**C**) Expression of major histocompatibility complex (MHC) class II and (**D**) activation markers CD80 on murine DCs stimulated for 24 h with either lipopolysaccharide (LPS; dose equivalent to that detected in WISIT conjugate preparations), the WISIT-conjugate vaccine CW-type 1, or the respective conventional peptide-protein conjugate CC-type 1 by flow cytometry (identification of DCs was performed based on CD11c expression) ** *p* < 0.01; *** *p* < 0.001 (exposure with CW-type 1 vs. LPS). Error bars indicate the mean ± SEM of one representative experiment performed in triplicates. Statistical differences were evaluated by one-way ANOVA and Tukey’s multiple comparison test. ** *p* < 0.01; *** *p* < 0.001 (exposure with CW-type 1 vs. LPS and CW-type 1 vs. CC-type 1). ns: No significant difference after exposure to conventional vaccine CC-type 1 vs. LPS. (**E**) Representative histogram overlays of DC CD80 (top) and MHC II (bottom) reactivity in response to vaccines as indicated in the legend insert.

To determine the biological activity of vaccine candidates, two assays were used. First, we analyzed binding to the cognate β-glucan pattern recognition receptor (PRR) dectin-1. Non-peptide coupled, activated CLECs as well as CW-type 1 and conventional CC-type 1 vaccines were assessed for binding to dectin-1, using a competitive ELISA. The comparison of CW-type 1 and conventional CC-type 1 vaccines showed that conventional peptide-protein conjugate vaccines do not interact with the β-glucan receptor (Figure 2A). Activated CLEC/β-glucan displayed high dectin-1 binding capacity that was slightly reduced after peptide coupling (CW-type 1). Similarly, four additional CW-type candidate vaccines were assessed. All additional CW-type candidates retained high and comparable dectin-1 binding, albeit slightly reduced compared to non-coupled β-glucan moieties (Figure 2B).

To demonstrate that WISIT vaccines targeting α-syn are not only binding to PRRs, but also exert biological function in their target cells, in vitro DC activation was measured. Murine GM-CSF generated DCs were stimulated in vitro with CW-type 1 as well as with a conventional CC-type 1 protein conjugate vaccine as a benchmark. Flow cytometry-based analysis of DC activation markers revealed significantly increased expression levels of CD80 and MHCII, indicative of DC maturation and activation (Figure 2C,D). The expression levels achieved were significantly higher than the effects observed by equivalent doses of LPS, contained within the conjugate preparations (Figure 2C,D; *p* < 0.001 and *p* < 0.01). The comparison to benchmark vaccines also revealed a significant increase in expression for both markers following CW-type 1 exposure (Figure 2C,D, *p* < 0.001 and *p* < 0.01). No significant increase in expression for MHCII or CD80 was observed for DCs stimulated with the CC-type 1 benchmark-type vaccine (Figure 2C,D).

### 3.4. WISIT PD Vaccines Elicit Higher Immune Responses Compared to Conventional PD Vaccines

**Figure 3:**
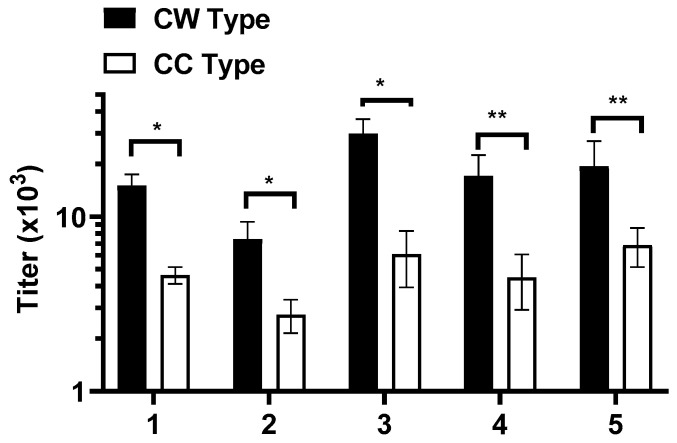
Target-specific immune response induced by WISIT vaccines. Titers of antibodies specific to recombinant full-length α-syn were detected in plasma collected after the third immunization with indicated immunogens by ELISA. WISIT-vaccines (CW type 1–5) induced significantly higher responses than conventional vaccines (CC type 1–5) adjuvanted with alum. Error bars indicate the mean values of antibody titers ± SEM of *n* = 10 animals per group. Statistical differences were evaluated by one-way ANOVA and Tukey’s multiple comparison test (* *p*  <  0.05; ** *p* < 0.01).

**Figure A1:**
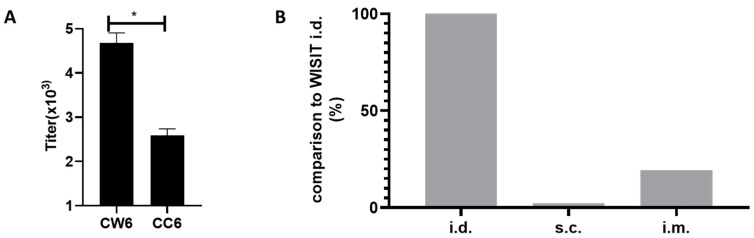
Target-specific immune response induced by WISIT-PD vaccine candidate 6 and optimal application route for WISIT CW-type 1. (**A**) Titers of antibodies specific to recombinant full-length α-syn were detected in plasma collected after the third immunization with indicated immunogens by ELISA. CW-type 6 induced significantly higher responses than CC-type 6 adjuvanted with aluminum. Error bars indicate the mean ± SEM of *n* = 6 animals per group. Statistical differences were evaluated by an unpaired *t*-test. * *p* < 0.05 (**B**) Comparative analysis of the antibody response in different immunization routes. Titers of antibodies specific to recombinant full-length α-syn were detected in plasma collected after the third immunization by ELISA. Values are expressed as percentage of anti α-syn titer after i.d. application (anti α-syn titer value following i.d. application corresponds to 100%). i.d.: Intradermal application; s.c.: Subcutaneous application; i.m.: Intramuscular application. Results of one representative experiment with *n* = 6–10 animals/group.

Next, we analyzed the induction of anti-α-syn protein-specific antibodies following three biweekly immunizations of BALB/c mice with WISIT and conventional PD-vaccines by plasma ELISA. WISIT vaccines were specifically designed to target DC in the skin. Consequently, a comparative analysis of the antibody response elicited by immunization using different immunization routes showed i.d. application to be the most effective route at generating a substantial antibody response for WISIT vaccine candidates (Appendix A Figure A1B). Next, i.d. immunization of WISIT vaccine candidates (CW-type 1–6) was compared to immune responses induced by the respective conventional benchmark vaccines (CC-type 1–6; applied s.c., aluminum adjuvanted). Plasma ELISA analysis showed that vaccination with CW-type vaccines resulted in the induction of significantly higher titers towards the α-syn protein compared to conventional CC-type vaccines (Figure 3 and Appendix A Figure A1A).

The data show that the *C*-terminal α-syn region aa 110–130 was the most immunogenic region tested in the study. Other domains were slightly less immunogenic; however, they still induced an immune response with similar characteristics (Appendix A Figure A1A). Hence, we focused on CW-type candidates 1–5 for further studies.

### 3.5. Antibodies Elicited by WISIT PD Vaccines Show Greater Binding and Selectivity to α-Syn Aggregates Than Conventional PD Vaccines

Besides the total amount of antibodies elicited by a vaccine, the stability of antibody–antigen complexes or resilience against disruptive factors, as well as the selectivity of generated antibodies for aggregated α-syn, are important parameters for evaluating the potential effect of a PD vaccine.

**Figure 4:**
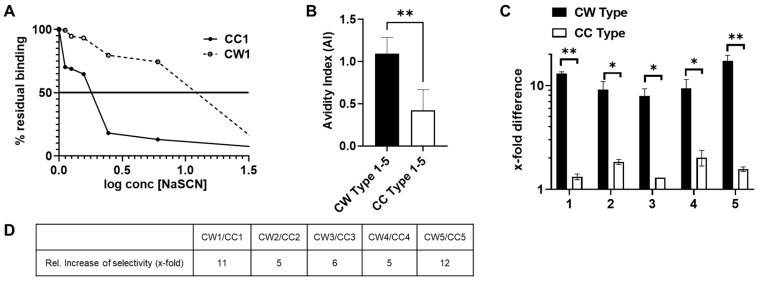
Avidity and selectivity of antibodies induced by WISIT vaccines. Investigation of the stability of α-syn-antibody complexes induced by indicated immunogens after challenging with different concentrations of the chaotropic agent sodiumthiocyanate (NaSCN) showing that WISIT-induced antibody-α-Syn complexes (CW type 1–5) were more stable than conventional vaccine-induced α-Syn complexes (CC type 1–5). (**A**) Residual binding towards α-syn filaments for CW type 1- and CC-type 1 induced antibodies after challenging with increasing concentrations of chaotropic thiocyanate ions (0.25 M to 3 M) and (**B**) avidity indexes determined. Error bars indicate the mean ± SEM of two different experiments performed in triplicate. Statistical differences were evaluated by one-way ANOVA and Tukey’s multiple comparison test (** *p* < 0.001). (**C**) Analysis of selectivity of the antibodies induced for α-syn filaments compared to monomeric α-syn by inhibition ELISA. Error bars indicate the mean ± SEM of three different experiments performed in triplicate. Statistical differences were evaluated by one-way ANOVA and Tukey’s multiple comparison test * *p* < 0.05; ** *p* < 0.01. (**D**) Fold difference in selectivity for α-syn filaments is depicted. Relative increase in selectivity shows the fold improvement of CW-type induced vs. CC-type induced Abs.

To estimate the stability of the antigen–antibody complexes, we measured their resistance against the chaotropic agent NaSCN [46,47]. This method is based on immunosorbent assays where the preformed antibody–antigen complex is transiently exposed to high molar concentrations of a chaotropic agent. The resistance to thiocyanate elution was used as a measure of avidity, and an index (i.e., the avidity index), representing 50% of effective antibody binding, was used to compare different sera. The residual binding analysis for CW1 relative to CC1-induced antibodies showed approximately four times higher avidity for α-syn filaments (Figure 4A). We found that WISIT PD vaccine candidates CW-type 1–5 induced antibody-α-syn complexes were significantly more stable than those formed by CC-type 1–5-induced antibodies (Figure 4B and Table 2).

**Figure A2:**
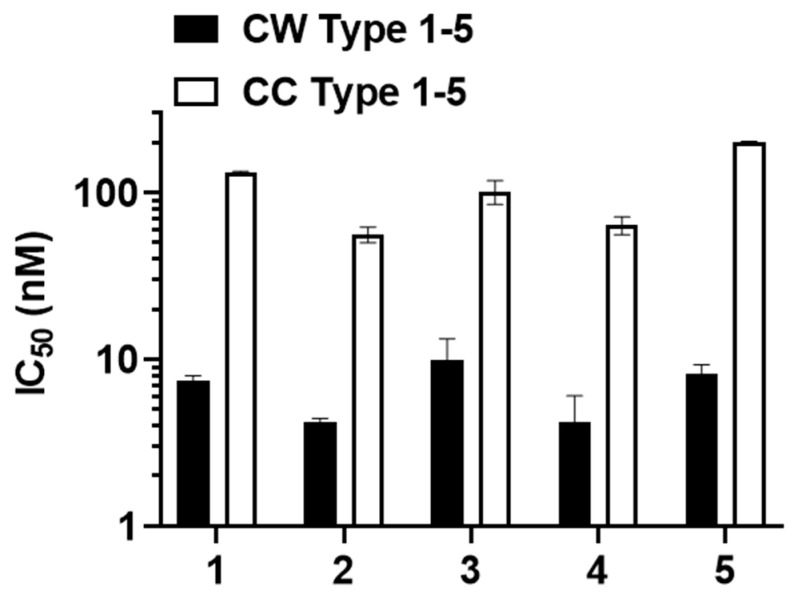
Selectivity of antibodies induced by WISIT vaccines. Determination of IC50 values for α-syn filaments by inhibition ELISA of induced antibodies. Error bars indicate the mean ± SEM of three different experiments performed in triplicate.

We then checked if the increased avidity associated with WISIT PD vaccine candidates is also accompanied with higher selectivity towards aggregated α-syn (Figure 4C,D). Indeed, a higher selectivity to α-syn filaments for CW-type 1–5 vaccine-induced antibodies was confirmed by inhibition ELISA, with average IC_50_ values towards the α-syn filament of 8 nM ± 1.5 for CW-type vaccine candidate-induced antibodies and 198 nM ± 6.5 for CC-type vaccine-induced antibodies (see Appendix A Figure A2). Interestingly, all CW-type vaccine candidates tested induced antibodies with highly increased selectivity towards α-syn filaments compared to α-syn monomers (between 8–18-fold higher selectivity towards α-syn filaments). In contrast, none of the conventional benchmark vaccines tested induced antibodies with more than 2-fold higher selectivity towards α-syn filaments. Thus, the relative improvement in selectivity towards α-syn filaments by antibodies induced by CW-type vaccines as compared to benchmark vaccines was between 5- and 12-fold.

**Figure A3:**
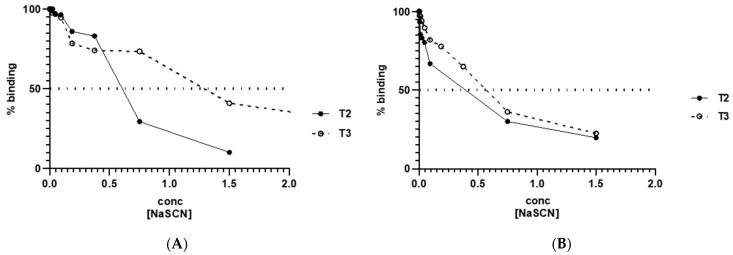
Affinity maturation of the α-syn-specific antibody response after immunization with CW-type 1 and CC-type 1 conjugate vaccines. Investigation of the stability of α-syn-antibody complexes induced two weeks after the second (T2) or two weeks after the third immunization (T3) after challenging with different concentrations of the chaotropic agent sodiumthiocyanate (NaSCN) showing that CW-type 1-induced antibodies are undergoing affinity maturation over the course of all three vaccinations. Residual binding towards α-syn filaments for (**A**) CW-type 1- and (**B**) CC-type 1-induced antibodies after challenging with increasing concentrations of chaotropic thiocyanate ions (0.25 M to 3 M). Results of one representative experiment performed in triplicates.

Given these differences in antibody quality, we started to characterize the immune responses of the two vaccine types with respect to the affinity maturation of the antibody response. After the first two vaccinations, the immune response was still comparable in terms of affinity. However, additional immunization revealed that the antibody response of the classical CC-type conjugate vaccines did not show a further increase in affinity, whereas the affinity of the CW-type-induced antibodies doubled with the third vaccination (Appendix A Figure A3).

### 3.6. WISIT Vaccine Candidates Inhibit α-Syn Aggregation In Vitro

**Figure 5:**
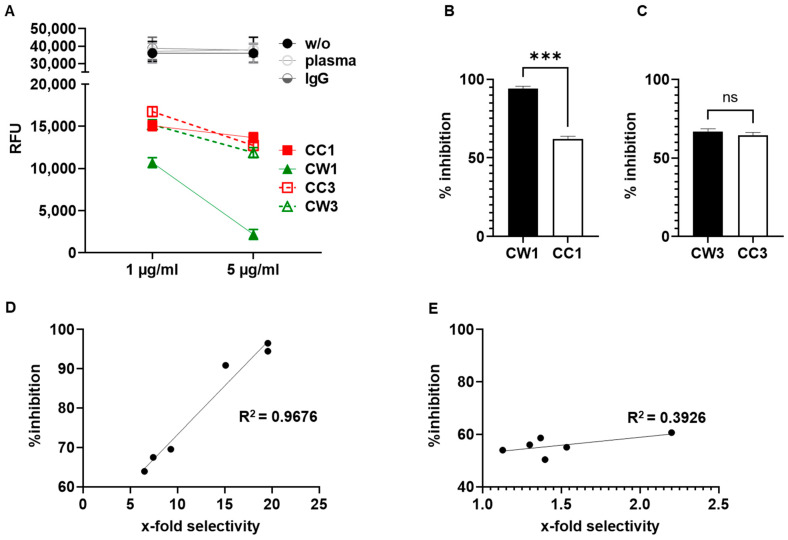
Antibodies induced by WISIT vaccines inhibit α-syn aggregation in vitro as a function of their selectivity for α-syn filaments. Inhibition of α-syn aggregation in the presence of vaccine-induced antibodies. (**A**) Thioflavin T (ThT) aggregation assay was performed in the presence of CW-type and CC-type vaccine-induced antibodies as well as IgG control antibodies or equivalent amounts of plasma from untreated mice. Data are shown as relative fluorescence units (mean RFU ± SEM of triplicates) of an in vitro aggregation assay after 72 h. (**B**,**C**) Percent inhibition of α-syn aggregation in the presence of 5 µg/mL antibodies at 72 h for (**B**) CW-type 1- and CC-type 1-induced antibodies; and (**C**) for CW-type 3 and CC-type 3 vaccine-induced antibodies. (**D**,**E**) Correlation between antibody selectivity and inhibitory capacity (% inhibition of α-syn aggregation) for (**D**) CW-type vaccines (r = 0.9838; CI (95%) 0.8547 to 0.9983, *p* = 0.0004, and R^2^ = 0.9679), and for (**E**) CC-type vaccines (r = 0.6266, CI (95%) 0.3764 to 0.9534, *p* = 0.1831 and R^2^ = 0.3926); ns: Not significant. Error bars indicate the mean ± SEM of three different experiments performed in triplicate. Statistical differences were evaluated by one-way ANOVA and Tukey’s multiple comparison test; ns: No significance; *** *p* < 0.001.

For functional characterization of the immune response induced by α-syn WISIT vaccine candidates, we assessed the capacity of WISIT-induced antibodies to inhibit α-syn aggregation using an in vitro α-syn aggregation and propagation assay [48,49,50]. Pre-formed α-syn fibrils (sPFF, Abcam) were mixed with α-syn monomers in the presence of titrated amounts of vaccine-induced or control antibodies (Figure 5). No effect on α-syn aggregation was observed using control IgG or mouse plasma only. In contrast, normalized amounts of CW-type vaccine- and conventional CC-type vaccine-induced antibodies could significantly inhibit or slow down aggregation (Figure 5A).

**Figure A4:**
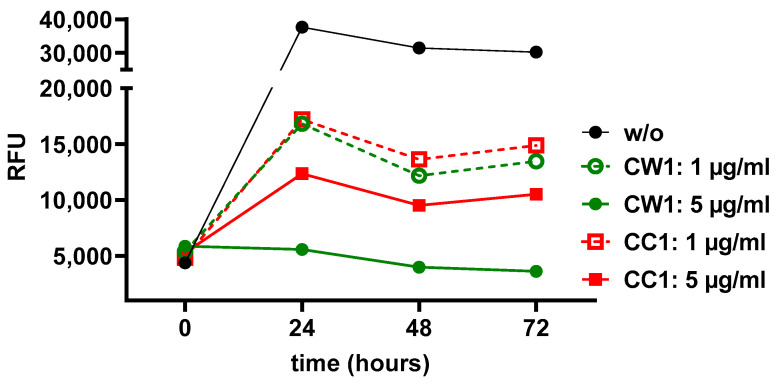
Inhibition of α-syn aggregation in the presence of vaccine-induced antibodies. Kinetic analysis of Thioflavin T (ThT) aggregation in the presence of CW-type and CC-type vaccine-induced antibodies. Data are shown as relative fluorescence units (mean RFU) of an in vitro aggregation assay at different time points (0–72 h).

CW-type 1 and CW-type 3-induced antibodies were found to inhibit further aggregation in a dose-dependent way during the 72 h testing period. In contrast, antibodies induced by conventional CC-type vaccines showed significantly weaker effects on aggregation inhibition. CW-type 1 induced highly aggregate selective antibodies, which in vitro induced a 94% reduction in aggregate formation compared to 58% induced by antibodies generated by the benchmark CC-type 1 vaccine (Figure 5, *p* < 0.001; kinetic of Thioflavin T (ThT) aggregation is shown in Appendix A Figure A4). Similarly, the CW-type 3 vaccine induced fewer aggregate selective antibodies, which showed a less pronounced in vitro inhibition effect as compared to CW-type 1.

As shown in Figure 5D, the selectivity of the vaccine-induced antibodies towards aggregated α-syn strongly correlated with their capacity to inhibit α-syn aggregation in vitro (Pearson r = 0.9838; CI (95%) 0.8547 to 0.9983, *p* = 0.0004, and R^2^ = 0.9679). In contrast, CC-type vaccine-induced antibodies showed only a weak positive correlation (Figure 5E, Pearson r = 0.6266, CI (95%) −0.3764 to 0.9534, *p* = 0.1831 and R^2^ = 0.3926). This analysis further supports that CC-type and WISIT CW-type vaccines have different immunological properties and induce antibody types with distinct binding characteristics towards α-syn aggregates.

### 3.7. WISIT Vaccine CW-Type 1 Inhibits Propagation of Synucleinopathy In Vivo

**Figure 6:**
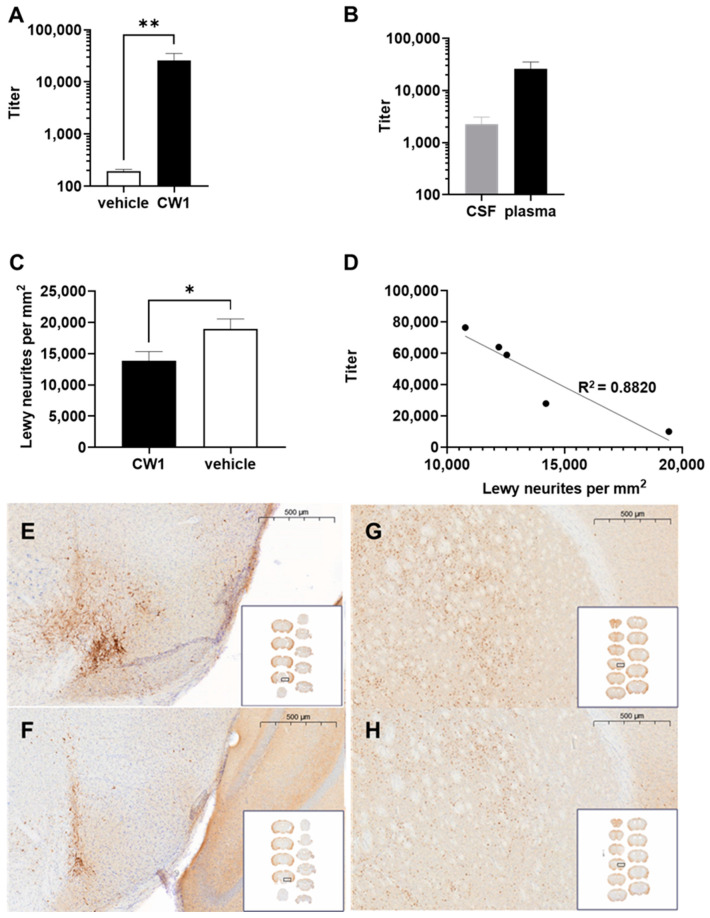
CW-type-induced antibodies inhibit α-syn aggregation in a PFF model in vivo. C57BL/6 mice stereotactically injected in the right substantia nigra with recombinant α-syn PFFs were immunized four times using CW-type 1 vaccine or unconjugated CLEC as control starting on the day of PFF inoculation. Plasma was collected after the third immunization. Brains, plasma, and CSF were harvested 126 days post-PFF inoculation. Plasma titer of antibodies specific to the peptide used for immunization (**A**) collected two weeks after the third immunization on day 126. (**B**) Comparison of CSF and plasma titers of antibodies specific for the B cell peptide of the vaccine at day 126. (**C**) Analysis of phosphor-S129 α-syn-positive aggregates over all brain areas in CW-type 1 vaccinated and CLEC-treated mice. (**D**) Correlation between antibody response and the level of synucleinopathy in CW-type 1 recipients (r = −0.9391; CI (95%) −0.9961 to −0.3318, *p* = 0.0179, and R^2^ = 0.882). (**E**–**H**) Representative pSer129 α-syn staining in the injected brain hemisphere at the level of (**E**,**F**) the substantia nigra and (**G**,**H**) the striatum. (**E**,**G**) Vehicle-treated mice and (**F**,**H**) CW-type 1 treated mice following PFF injection. Error bars indicate the mean ± SEM of *n* = 5–9 animals per group. Statistical differences were evaluated by an unpaired *t*-test; ** *p* < 0.01; * *p* < 0.05.

To determine if WISIT vaccine candidates were able to inhibit α-syn fibril formation in vivo, a proof-of-concept experiment for WISIT vaccine CW-type 1 was initiated using an established seeding model for synucleinopathies [44]. In this model, C57BL/6 mice are stereotactically injected with α-syn pre-formed fibrils (PFFs) at the level of the right substantia nigra, subsequently causing widespread synucleinopathy, characterized specifically by phosphosynuclein immunopositive Lewy-like neurites and intracytoplasmic aggregates along anatomical connections. Animals were immunized four times at weeks 0, 2, 4, and 10 with CW-type 1, or non-conjugated CLEC as the control, starting the first immunization on the day of PFF inoculation. Then, 126 days post-PFF-injection, animals were sacrificed, and brains were analyzed for the presence of phosphor-S129 α-syn-positive aggregates in selected brain areas including the cerebral cortex, striatum, thalamus, substantia nigra, and brainstem. Analysis of the ensuing immune response was performed using plasma and CSF obtained at the time of sacrifice. High-antibody titers against the injected peptide were detected in the plasma of CW-type 1-treated animals. In contrast, no signal above the background could be detected in the CLEC-only-treated group (Figure 6A). Analysis of the anti-peptide titer in CSF also showed a high level of CW-type 1-induced antibodies, whereas no signal above background was detectable for the vehicle-treated animals (Figure 6B). Immunohistochemistry of brain sections showed high numbers of phosphor-S129 α-syn-positive aggregates throughout all analyzed areas in the vehicle-treated group indicating a strong propagation of α-syn pathology. In contrast, synucleinopathy was significantly reduced in CW-type 1-vaccinated mice (Figure 6C,E,F). Of note, there was a strong and significant reciprocal correlation between the strength of the antibody response and the level of synucleinopathy in CW-type 1 recipients (Figure 6D).

## 4. Discussion

Immunotherapy targeting α-syn is the most promising approach to slow PD disease progression to date. Specific antibodies eliminating α-syn aggregates can be either passively introduced or induced by active vaccination. Several first-generation α-syn-immunotherapeutics are in early clinical development. However, substantial improvements in the core technology are needed to achieve lower toxicity, higher efficacy, and more economical production to enable scalability.

The α-syn WISIT vaccine platform is designed to effectively leverage skin DCs by i.d. vaccination to generate substantially stronger and more specific immune responses compared to conventional α-syn conjugate vaccines. The skin represents a rich source of potent DCs capable of mounting strong immune responses, which are not utilized by available PD vaccines. WISIT constructs are unique as the vaccine backbone itself comprises a DC-targeting/stimulating agent (β-glucan), fused to variable T-helper and α-syn B-cell peptide epitopes, making WISIT vaccines cheaper to produce and more flexible than existing vaccines by design. The WISIT constructs used in this study consist of a β-glucan conjugated to a promiscuous Th cell epitope, the pan DR epitope PADRE. They differ at the level of the α-syn-specific variable B cell epitopes, which define the specificity of the ensuing antibody response. Specifically, β-1,3-glucans interact with dectin-1 receptors on DC to stimulate DC migration and maturation [51]. By using β-glucan as the constant backbone whilst flexibly modifying the Th- or α-syn B cell epitopes, WISIT is constructed as a “Lego”-style vaccine platform with interchangeable units that are capable of leveraging skin immunity to improve the vaccine’s immunogenicity.

Here, we show that PD WISIT vaccine candidates are superior to conventional conjugate vaccines in inducing antibodies that interfere with α-syn aggregation and propagation in vitro. Preliminary in vivo studies demonstrate the WISIT vaccine CW-type 1 to prevent the propagation of α-syn pathology in an in vivo seeding model for synucleinopathies. The efficacy of WISIT vaccines was found to rely on two main immunological features. First, they are more immunogenic than conventional conjugate vaccines and induce higher antibody titers specific to the target α-syn. Second, antibodies elicited by WISIT vaccines are characterized by a dramatically improved selectivity/avidity for α-syn aggregates, the pathological α-syn moiety in PD. The functional relevance of the high antibody selectivity is supported by our finding of a strong and direct correlation between the antibody selectivity for α-syn filaments and their ability to inhibit α-syn aggregation in vitro. These findings are further in line with the results of a recent study by Höllerhage et al. demonstrating that the protective efficacy of anti-α-syn antibodies is a function of the stability of the antibody/α-syn complexes formed [52]. Using a neuronal co-culture model, in which α-syn species are released from α-syn-overexpressing cells and induce toxicity in a priori healthy cells, they found other characteristics of the antibodies, such as their α-syn epitope-binding domain and the degree of specificity of the antibodies, to be irrelevant for the therapeutic efficacy of a given α-syn antibody.

The demonstration that WISIT vaccines bind to dectin-1 and can activate DCs supports the hypothesis that they act by targeting skin-resident DCs. This is further corroborated by our finding that their administration to the dermal compartment is more efficacious than application to subcutaneous or muscle tissue in inducing target-specific antibody responses. The view that WISIT constructs act via DCs resident to the dermal compartment is in line with previous reports providing direct evidence for DC targeting via fluorescence labeling of constructs [37,53].

DC targeting is a well-known strategy for increasing the magnitude and quality of the antibody response [54]. Indeed, the fundamental difference between available peptide conjugate vaccines and skin-DC-targeted WISIT-type vaccines with respect to their antibody response, is the higher affinity for α-syn and higher selectivity for aggregated forms of α-syn induced by the WISIT vaccines. While affinity maturation upon administration of conventional vaccines is blocked after the second vaccination, affinity doubled upon the third WISIT application. Initial dose titration studies additionally document the difference between the vaccine types as there is no plateau with WISIT, and more extensive studies with a wider dose range are currently ongoing.

Six different peptides (7–12 aa) covering different α-syn domains (*N*- and *C*-terminal) were tested in the current study using the WISIT vaccine platform. Superiority over state-of-the-art conjugate vaccines was proven to be independent of the epitope-length used for immunization. Thus, peptides that are too short to contain potential MHC-I or MHC-II epitopes can be effectively used, excluding the danger of inducing α-syn specific T-cells in vivo.

## 5. Conclusions

The data presented in this study demonstrate the α-syn WISIT vaccine technology to be superior to conventional conjugate vaccines regarding both the titer of antibodies induced to an α-syn B cell epitope and also the avidity/selectivity of the antibodies elicited. In the case of our α-syn targeting vaccines, this translates into a functional benefit since induced antibodies inhibit α-syn aggregation and propagation of pathology more efficiently than antibodies triggered by their conventional counterparts. It will be interesting to explore whether the improved immunological and functional characteristics of WISIT vaccines will translate to humans. Given the fact that both backbone elements, PADRE and β-glucans, demonstrated activity in humans [55,56,57,58], WISIT technology may deliver novel α-syn-based vaccine candidates capable of modifying the course of the disease in PD patients.

## Figures and Tables

**Table 1:** Composition of WISIT-type and conventional vaccine-type candidates. The number in the name identifies the B cell peptide. The other components of the individual vaccines used are detailed. CW: WISIT-type candidate; CC: Conventional vaccine-type candidate.

Name	B Cell Epitope	T Cell Epitope	Sugar Moiety or Adjuvant
WISIT Vaccine Candidates			
CW-type 1	α-syn 110–130 (7-mer)	PADRE	β-glucan/CLEC
CW-type 2	α-syn 110–130 (8-mer)	PADRE	β-glucan/CLEC
CW-type 3	α-syn 110–130 (10-mer)	PADRE	β-glucan/CLEC
CW-type 4	α-syn 110–130 (11-mer)	PADRE	β-glucan/CLEC
CW-type 5	α-syn 110–130 (12-mer)	PADRE	β-glucan/CLEC
CW-type 6	α-syn 1–10 (8-mer)	PADRE	β-glucan/CLEC
Conventional Vaccine-Type Candidates			
CC-type 1	α-syn 110–130 (7-mer)	CRM 197	aluminum oxyhydroxide
CC-type 2	α-syn 110–130 (8-mer)	CRM 197	aluminum oxyhydroxide
CC-type 3	α-syn 110–130 (10-mer)	CRM 197	aluminum oxyhydroxide
CC-type 4	α-syn 110–130 (11-mer)	CRM 197	aluminum oxyhydroxide
CC-type 5	α-syn 110–130 (12-mer)	CRM 197	aluminum oxyhydroxide
CC-type 6	α-syn 1–10 (8-mer)	CRM 197	aluminum oxyhydroxide

**Table 2:** CW-type and CC-type vaccine-induced antibody avidity index comparison.

	CW-Type	CC-Type	Factor
Candidate 1	1.1	0.25	4.4
Candidate 2	0.75	0.3	2.5
Candidate 3	1.2	0.4	3
Candidate 4	1.2	0.4	3
Candidate 5	1.2	0.3	4

## Data Availability

The data presented in this study are available on request from the corresponding author.
